# CX-5461 Inhibits Pancreatic Ductal Adenocarcinoma Cell Growth, Migration and Induces DNA Damage

**DOI:** 10.3390/molecules24244445

**Published:** 2019-12-04

**Authors:** Btissame El Hassouni, Giulia Mantini, Benoît Immordino, Godefridus J. Peters, Elisa Giovannetti

**Affiliations:** 1 Department of Medical Oncology, Cancer Center Amsterdam, Amsterdam UMC, VU University Medical Center (VUmc), 1081 HV Amsterdam, The Netherlands; b.elhassouni@amsterdamumc.nl (B.E.H.); g.mantini@amsterdamumc.nl (G.M.); benoit-immordino@hotmail.com (B.I.); gj.peters@amsterdamumc.nl (G.J.P.); 2 Department of Biochemistry, Medical University of Gdansk, 80-210 Gdańsk, Poland; 3 Cancer Pharmacology Lab, AIRC Start Up Unit, Fondazione Pisana per la Scienza, 56017 Pisa, Italy

**Keywords:** CX-5461, Pol I, PDAC, DNA damage

## Abstract

Background: Inhibition of ribosome biogenesis has recently emerged as a promising strategy for the treatment of metastatic tumors. The RNA polymerase I inhibitor CX-5461 has shown efficacy in a panel of cancer types and is currently being tested in clinical trials. However, further preclinical studies to unravel molecular mechanisms underlying the activity of this drug are warranted. Methods: In this study, we have investigated the effects of CX-5461 on cell growth and migration of pancreatic cancer cells by the sulforhodamine-B and wound healing assay, respectively. Furthermore, we assessed the expression of epithelial-to-mesenchymal transition (EMT) genes by qRT-PCR, while protein expression of DNA damage marker phospho-H2A.X was studied by Western blot and immunofluorescence. Results: CX-5461 inhibits pancreatic cancer cell growth in the nanomolar range and inhibits the migratory capability of the cells. Additionally, CX-5461 induced expression of EMT factor SNAI1 and caused DNA double-strand breaks as measured by increased expression of phospho-H2A.X. Conclusion: This study demonstrated that CX-5461 is active against pancreatic cancer cells and modulation of EMT factors, as well as increased expression of phospho-H2A.X, support further pre-/clinical investigations, including the analyses of these markers.

## 1. Introduction

Pancreatic cancer (PC) is a highly metastatic disease with a poor five-year survival of less than 10% [1]. Patients diagnosed with PC often present an advanced disease stage limiting their treatment options to chemotherapy. However, patients frequently do not respond to the treatment or develop resistance to the current regimens. Therefore, novel treatment options are warranted. 

Ribosome biogenesis is a process that is upregulated in cancer, where it contributes to increased protein production facilitating cell proliferation [2]. Exploiting the ribosome biogenesis pathway [3], and more specifically, RNA polymerase I (pol I) inhibition for cancer treatment, has shown promising results in recent years [4,5]. Notably, an enhanced ribosome biogenesis rate has been found in pancreatic cells of patients with chronic pancreatitis [6], which suggests a possible therapeutic window. Additionally, a computational analysis of 34 pancreatic ductal adenocarcinoma samples highlights the important role of ribosome biogenesis genes in PDAC progression [7]. 

CX-5461, a small molecule synthesized by Cylene Pharmaceuticals [5], is the first Pol 1 inhibitor that is being tested in a phase 1 dose-escalation clinical trial for hematological cancers [8]. CX-5461 blocks proliferation in various cancer types by inhibiting Pol I and is capable of inducing p53-independent senescence and pro-death autophagy [5]. In neuroblastoma cells, CX-5461 activates p53 leading to cell cycle arrest or apoptosis [9]. It also suppressed MycN expression in cells and led to decreased tumor growth in xenograft nude mice. Moreover, CX-5461 can function as a G-quadruplex stabilizer with increased toxicity in BRCA-deficient or PARP inhibition-resistant cancer cells [10]. Mechanistically, CX-5461 blocks replication forks and induces DNA gaps or breaks. CX-5461 is a potential radiosensitizer, since it increased the sensitivity of low dose single X-ray exposure in cervical CaSki cancer cells leading to cell apoptosis, autophagy, and senescence [11]. Therefore, CX-5461 shows great potential as a future treatment option. 

In this study, we show the effect of CX-5461 on cell growth and the migration of PC cells and primary cell culture. Moreover, we report its effect on epithelial-to-mesenchymal transition markers and the induced DNA damage. 

## 2. Results

### 2.1. CX-5461 Inhibits Pancreatic Cancer Cell Growth in Nanomolar Range 

**Figure 1:**
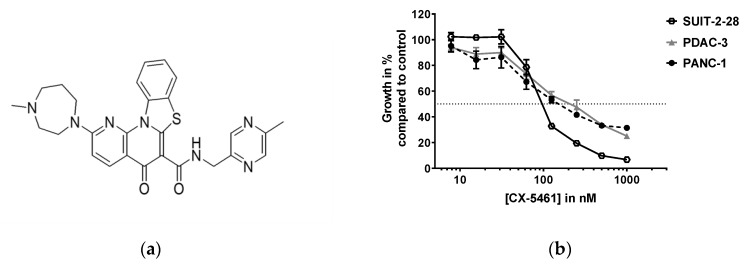
CX-5461 inhibits pancreatic cancer cell growth in the nanomolar range. (**a**) Chemical structure of CX-5461 designed with ChemDraw. (**b**) Growth inhibition curves relative to untreated control of pancreatic cancer cells SUIT-2-28 (connecting black line), PDAC-3 (grey line) and PANC-1 (dashed black line) treated for 72 h with CX-5461. The dashed line (y = 50) indicates fifty percent growth inhibition.

Growth inhibition studies were performed in order to determine the sensitivity to the small molecule CX-5461 (Figure 1a) in different preclinical models of PC. Two PC cell lines, epithelial SUIT-2-28 [12], mesenchymal PANC-1 [12], and the primary cell culture PDAC-3 were exposed to CX-5461 and showed similar sensitivity, within the nanomolar range (Figure 1b). The concentrations that caused inhibition of 50% of the cell growth (IC_50_) are listed in Table 1, together with the doubling time of the cells. The doubling time is calculated based on the optical density (OD) of the untreated control cells after 72 h divided by the OD after 24 h of cell attachment (D0), and by correcting for the background and determining the number of doublings based on the exponential growth. Both SUIT-2-28 and PDAC-3 have a doubling time of less than 20 h, whereas the PANC-1 cells divide slower (28.4 ± 1.91 h). Previous data showing that inhibition of ribosome is higher in rapidly proliferating cancer cells [5] seem, therefore, to reflect in a higher IC_50_ value for the PANC-1 cells. 

### 2.2. CX-5461 Inhibits PANC-1 Cell Migration 

PC has one of the highest rates of metastasis among solid cancers, and a better understanding of the anti-migratory effects of new drugs is essential in order to provide more effective tools for therapeutic purposes [13].

The high sensitivity of the tested PC cells to CX-5461 and previous findings suggesting that ribosome biogenesis contributes to metastatic cancer progression [14] prompted us to investigate the effect of this drug on cell migration, using the wound healing assay. 

In order to eliminate the possible confounding effect of cell proliferation on the migration results, the cells with the highest doubling time were used (PANC-1). A concentration of 1.5 μM (7.5 times the IC_50_) of CX-5461 was tested, because of the shorter drug exposure time compared to the growth inhibition studies, lasting 72 h. Of note, this concentration was also selected since it is within the “therapeutic window” for effective exposure, considering the AUC and Cmax values reported in clinical studies [8]. Since SUIT-2-28 and PDAC-3 cells did not produce reproducible confluent layers, necessary to evaluate scratches and to perform a migration assay, we could not produce reliable data with these two cell lines.

**Figure 2:**
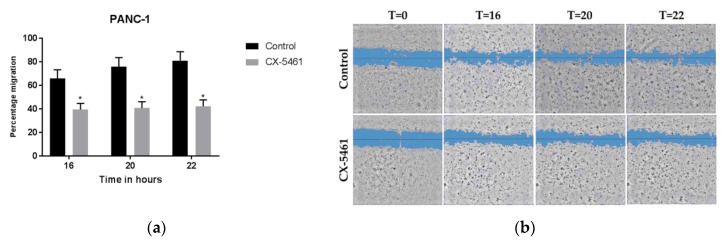
CX-5461 inhibits PANC-1 cell migration. (**a**) Migratory inhibition by CX-5461 in PANC-1 cells was shown as percent of migration calculated based on t = 0 time point, with control (untreated) cells (black bars) and 1.5 μM CX-5461 (grey bars), significance * *p* < 0.05, t-test (**b**) Representative images of the PANC-1 scratch areas in control (untreated cells) and in cells treated with CX-5461 over time.

Interestingly, CX-5461 treatment inhibited cell migration significantly at 16, 20, and 22 h after treatment, resulting in 40% cell migration in treated cells compared to 65% in untreated cells after 16 h (Figure 2a,b). Notably, at the more advanced time points of 20 and 22 h, the cell migration of CX-5461 treated cells remained 40%, compared to increased migration of the untreated cells. 

### 2.3. CX-5461 Induces mRNA Expression of EMT Markers 

The migratory capability of cells is often linked to an altered expression of epithelial to mesenchymal transition (EMT) or mesenchymal to epithelial transition (MET) markers. Therefore, we investigated the effect of CX-5461 on the mRNA expression levels of snail (SNAI1), slug (SNAI2), E-cadherin (CDH1), N-Cadherin (CDH12), vimentin (VIM), and matrix metalloproteinase 9 (MMP9). The cells were exposed to CX-5461 for 24 h.

**Figure 3:**
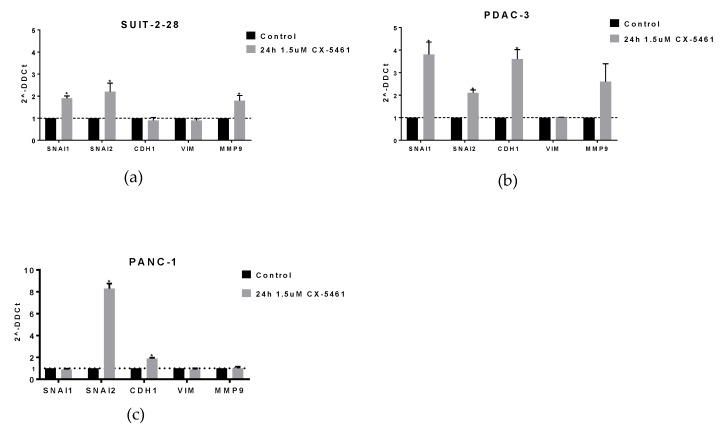
Effect of CX-5461 on the mRNA expression of EMT markers. (**a**) SUIT-2-28 (**b**) PDAC-3 (**c**) PANC-1. Significance * *p*-value < 0.05. The experiments were conducted in duplicate or triplicate and repeated at least twice (unpaired t-test).

Interestingly, we observed slightly different effects in the different cellular models. For instance, SNAI1 and SNAI2 were increased in SUIT-2-28 and PDAC-3 (Figure 3a,b), whereas SNAI1 and MMP9 remained unaltered in PANC-1 after drug exposure (Figure 3c). Moreover, CX-5461 treatment increased the expression of CDH1 in PDAC-3 and PANC-1 cells, whereas no alteration was observed in SUIT-2-28. These results suggest that CX-5461 hampers the cells in the epithelial phenotype, but further studies should investigate other aspects underlying the relative contributions of inhibition of Pol I in these PDAC cells considering the effects on migration versus EMT. 

### 2.4. DNA Damage Induced by CX-5461

Previous studies showed that CX-5461 has selective lethality in tumors deficient in DNA damage repair [10]. In the present study, induction of DNA double-strand breaks was demonstrated by phosphorylation of histone H2A.X at serine residue 139. Moreover, actin was stained to visualize the shape of the cells. 

**Figure 4:**
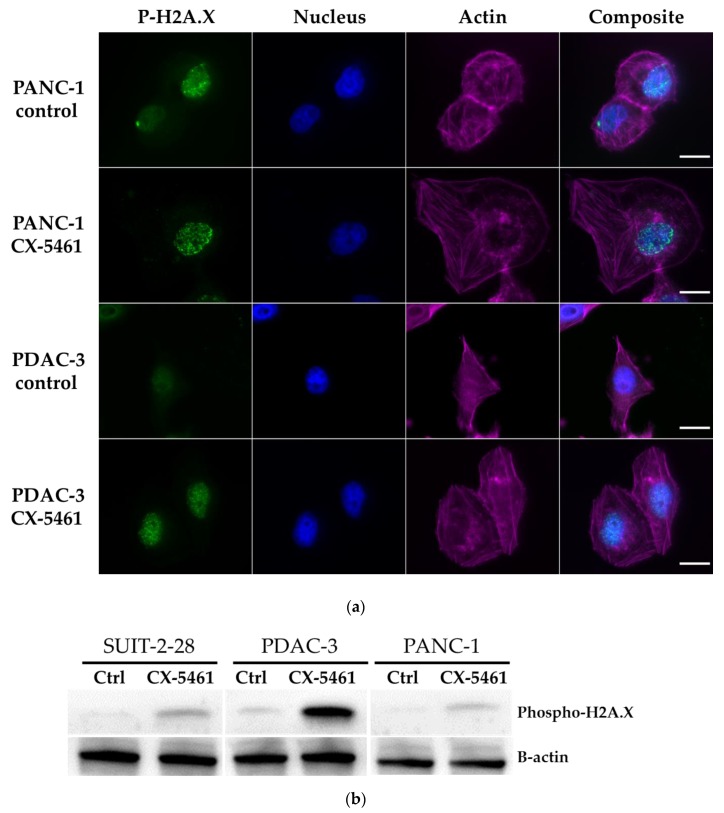
CX-5461 induces DNA damage. (**a**) Immunofluorescence staining of phospho-H2A.X (green), nucleus (DAPI, blue), actin (TRITC conjugated phalloidin, magenta) of control (untreated) cells or cells exposed to 1.5 µM CX-5461 for 24 h, with increased phospho-H2A.X staining in the CX-5461 treated cells. Fluorescence intensities of phospho-H2A.X were scaled to the same range for all conditions. Scale bar: 20 μm. (**b**) Increased phospho-H2A.X protein expression after 24 h treatment with 1.5 μM CX-5461.

In both PDAC-3 and PANC-1 cells, we found an increased expression of phospho-H2A.X after drug exposure by immunofluorescence, as shown in the representative images in Figure 4a. The increased protein expression of phospho-H2A.X after CX-5461 treatment was further confirmed by Western blot (Figure 4b). SUIT-2-28 cells showed a similar pattern as PANC-1 and PDAC-3 cells (data not shown).

### 2.5. The Effect of CX-5461 in 3D Spheroid Models 

Monolayer cytotoxicity and migration assays provide important data to evaluate drug activity, but three-dimensional (3D) spheroid models represent a useful platform for further identifying both the biological characteristics of tumor cells, and the drug sensitivity. Moreover, they can be a bridge between traditional 2D culture and animal experiments. 

**Figure 5:**
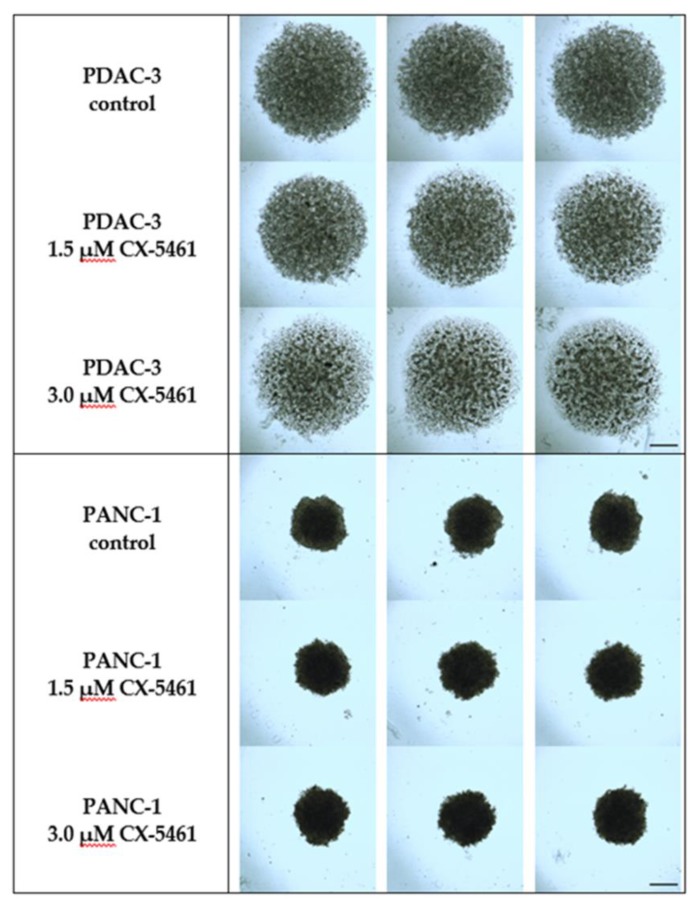
Effect of CX-5461on the integrity of PDAC-3 and PANC-1 spheroids after 96 h of treatment. Experiments were performed in at least triplicates and repeated twice. Scale bar: 500 μm.

The spheroids were exposed to 1.5 or 3.0 μM CX-5461 for 96 h. Figure 5 shows that PDAC-3 cells grown in the presence of CX-5461 were smaller and less dense compared to the untreated control spheroids. These findings suggest that CX-5461 might be active in more representative 3D models of primary cell cultures. However, CX-5461 did not have an effect on the size nor density of the PANC-1 spheroids. This might be explained by the fact that the control spheroid of the PDAC-3 cells was not as compact or dense as PANC-1. Previous studies showed that larger and denser spheroids have oxygen and nutrient gradients that often result in the formation of a necrotic core which might explain their resistance. Future studies should, therefore, evaluate if the spheroids resemble tumor cell physiology and how they should be used to increase the mechanistic understanding of anti-tumor drug activity.

### 2.6. Combination of CX-5461 and Gemcitabine Leads to An Antagonistic Effect 

Gemcitabine is one of the treatment options for patients with PC. Therefore, we investigated this drug at various concentrations in combination with the cell line specific IC_50_ of CX-5461. We observed a curve shift at the lower concentrations of gemcitabine for PANC-1 cells, but an overlap of the gemcitabine and gemcitabine with CX-5461 curves of PDAC-3 (Figure S1a,b). Based on the calculations performed with Calcusyn, this combination resulted in a combination index (CI) greater than 1.2 (Figure S1c), classifying the gemcitabine and CX-5461 interaction as antagonistic. Initial data with the SUIT-2-28 cells gave a similar pattern. Therefore, we did not proceed with this cell line. Further investigation of this interaction led to the finding that cytidine deaminase (CDA) mRNA expression was increased after CX-5461 treatment (Figure S1d), which may lead to increased degradation of gemcitabine. Moreover, in PDAC-3 cells exposed to CX-5461 we observed an increase of ribonucleotide reductase 2, which is a resistance mechanism of gemcitabine [15]. 

## 3. Discussion

In this study, we show that PC cells are sensitive to the small molecule CX-5461, in a nanomolar range, which is in line with previous studies [5]. Moreover, we showed that the migratory capability of the PANC-1 cells was inhibited, but this could not be correlated with the expression of EMT markers. The lack of vimentin modulation is in line with the results of Prakash and collaborators in NNuMG cells treated with CX-5461 [14]. However, we found an upregulation of SNAIL1 mRNA expression after CX-5461 exposure in PDAC-3 and SUIT-2-28 cells, whereas the previous study [14] shows only an increase of SNAIL1 in transforming growth factor β (TGF-β) stimulated cells treated with CX-5461. Furthermore, pro-mesenchymal marker SNAIL2 is the only marker upregulated in all three cell models, warranting further investigation. 

CX-5461 is capable of inducing DNA double-strand breaks as indicated by an increased formation of phospho-H2A.X in PC cells and in the primary cell culture PDAC-3, as reported before in U2OS cells and ovarian cancer cells [10,16]. 

The effect of CX-5461 on the spheroids was different in the two used cell lines but it could be ascribed to the difference in cell type, i.e., primary cell culture PDAC-3 and immortalized PANC-1. In vivo, CX-5461 had an antitumor effect against MIA PaCa-2 human PC, as well as neuroblastoma and melanoma xenografts [5,9]. In taxane-resistant triple-negative breast cancer PDX tumors, CX-5461 reduced tumor growth and showed selective lethality in BRCA1/2-deficient tumors [10]. 

Of note, many clinical trials evaluated gemcitabine in combination with potentially synergistic conventional chemotherapeutic agents, such as irinotecan, oxaliplatin, cisplatin, and 5-fluorouracil, but most of the following trials on these combinations have failed to show statistically significant results [17]. Moreover, combinations with tyrosine kinase inhibitors were negative, except for a minor effect of erlotinib. These findings should further prompt to consider different second-line therapies. Remarkably, recent studies showed positive results in maintenance studies, such as in the case of a phase II trial with sunitinib [18] and in the seminal POLO III trial with PARP inhibitors in BRCA-mutated patients treated in first-line with platinum-based therapies [19]. Considering our preclinical data and the fact that gemcitabine causes a different type of DNA damage, we reckon that CX-5461 should not be combined with gemcitabine. However, we speculate that CX-5461 might have more clinical value when used after first-line treatment, particularly in patients with aberrant DNA repair in combination with DNA damaging drugs, such as oxaliplatin or cisplatin, as was demonstrated earlier by Xu et al. [10]. Nevertheless, we are in urgent need of biomarkers that allow molecular monitoring to determine the proper therapy.

## 4. Materials and Methods 

### 4.1. Cell Culture 

The pancreatic cancer cell lines PANC-1 (ATCC^®^ CRL-1469), SUIT-2-28 [20] and primary cell culture PDAC-3 [21] were cultured in RPMI medium 1640 (catalog #21875034, Gibco, Waltham, MA USA) supplemented with 10% newborn calf serum (catalog #S0750-500, Biowest, Nuaillé, France). Cells were incubated at 37 ℃, 5% CO_2_ and tested frequently for mycoplasma contamination with the MycoAlert Mycoplasma Detection Kit (Cat no. LO LT07-705, Westburg, Leusden, The Netherlands). 

### 4.2. Chemicals 

CX-5461 (suppliers code HY-13323, MedChem Express, Monmouth Junction, NJ, USA) was solubilized in DMSO containing 0.1N HCl. 

### 4.3. Sulforhodamine B (SRB) Assay

The SRB assay was conducted as reported previously [22]. Briefly, cells were seeded at a density of 3000 cells per well in a flat-bottomed 96 well plate (VWR, Leicestershire, UK), left 24 h for attachment and subsequently treated with eight drug concentrations for 72 h. After cell fixation (5.6% TCA), washing steps, SRB staining (0.4% *w*/*v* SRB in 1% acetic acid), and resuspension in 10 mM Tris buffer, the optical density was measured at 490 and 540 nm on a BioTek plate reader (BioTek Instruments Inc., Winooski, VT, USA). 

### 4.4. Migration Assay 

PANC-1 cells were seeded in 96-well plates with a density of 30,000 cells per well to form a confluent monolayer overnight. Subsequently, the cells were scraped with a 96-well pin tool scratcher and detached cells were removed by washing steps of phosphate-buffered saline (PBS). Medium only or medium containing 1.5 µM CX-5461 was added to the wells and brightfield images were taken with software Universal Grab 6.3 digital (Digital Cell Imaging Labs, Keerbergen, Belgium) on a Leica DMI300B microscope (Leica Microsystems, Eindhoven, The Netherlands) at various time points. The obtained images were analyzed using the Scratch Assay 6.2 software (Digital Cell imaging Labs, Keerbergen, Belgium).

### 4.5. qRT-PCR 

Cells were treated with 1.5 μM CX-5461 or medium for 24 h and RNA was isolated according to the TRIzol reagent protocol (15596-026, ThermoFisher Scientific, Waltham, MA, USA). One microgram of RNA was then used for cDNA synthesis and subsequent PCR using the First-Strand cDNA synthesis kit (K1612, ThermoFisher Scientific, (Waltham, MA, USA).

### 4.6. Immunofluoresent Staining and Imaging

Cells were seeded on VWR 18 × 18 mm cover glasses (thickness 1.5) and incubated for 24 h for attachment. Subsequently, the cells were treated either with drug-free medium or medium containing 1.5 μM CX-5461 for 24 h. After three washing steps with PBS, cells were fixed with 200 μL 4% paraformaldehyde (PFA) (15710, Electron Microscopy Sciences, Hatfield, PA, USA) diluted in PBS for 10 min at room temperature (RT). After three other washing steps, cells were permeabilized 10 min at RT with 0.1% Triton x-100 (108643, Merck, Amsterdam, The Netherlands) diluted in PBS prior to overnight incubation at 4 °C with primary antibody Phospho-H2A.X (#2577, cell signaling, 1:50). Secondary antibody incubation was performed with Abberior STAR 488 (ST488, Abberior, Göttingen, Germany) and actin (TRITC conjugated phalloidin, Sigma-Aldrich, Zwijndrecht, The Netherlands) for 2 h at RT, followed by 15 min DAPI incubation. The coverslips were mounted in PBS and images were obtained on a widefield Zeiss Observer Z1 microscope (Zeiss, Jena, Germany) equipped with a CCD camera (EXI Aqua, QImaging, Surrey, BC, Canada). Illumination of samples was performed by an HXP 120 C lamp (Zeiss, Jena, Germany), and light was collected using a 63X oil immersion objective (NA = 1.4, Zeiss, Jena, Germany). The following filters were used: FITC (475/40 and 530/50 nm for excitation and emission filters, respectively), DAPI (365 and 445/50 nm for excitation and emission filters, respectively), and TRITC (545/25 and 605/70 nm for excitation and emission filters, respectively). Image processing was performed using ImageJ (NIH). 

### 4.7. Western Blot 

Whole-cell lysates were prepared from cells treated with 1.5 μM CX-5461 for 24 h or medium as control, by addition of cell lysis buffer (#9803, Cell signaling, Leiden, The Netherlands) diluted in demineralized water and supplemented with sodium orthovanadate (S6508, Sigma-Aldrich, Zwijndrecht, The Netherlands) and protease inhibitor cocktail (11697498001, Sigma-Alrich, Zwijndrecht, The Netherlands), followed by incubation on ice for 30 min. The samples were spun down at 16,000× *g* for 10 min at 4 °C and the supernatant was used to perform the Bio-rad protein estimation using the protein assay dye reagent concentrate (#500-0006, Bio-Rad, Veenendaal, The Netherlands). Finally, 50 μg protein samples were separated on Mini-Protean TGX^TM^ precast gels and transferred to PDVF membranes prior to target detection on the Uvitec using ECL^TM^ Prime Western blotting detection reagent (lot#13601176, GE Healthcare Bio-Sciences, Pittsburgh, PA, USA).

Antibodies: Primary, Phospho-Histone H2A.X (#2577, Cell signaling, 1:1000), β-actin (#4967S, Cell signaling, 1:2000). Secondary, Anti-rabbit IgG HRP linked (#7074, Cell signaling, 1:2000), Anti-mouse IgG HRP linked (#7076, Cell signaling, 1:2000).

### 4.8. Spheroid Formation 

Cells were seeded in ultra-repellent (7007, Costar, Washington, DC, USA) at a density of 30,000 cells/well and 10,000 cells/well for PDAC-3 and PANC-1 cells, respectively. After seeding, the plates were spun down at 200× *g* for 5 min at room temperature, followed by three-day incubation for solid spheroid formation. On day 3, either medium only or medium containing CX-5461 was added to the wells and the spheroids were imaged using Universal Grab (Digital Cell imaging Labs, Keerbergen, Belgium) on a Leica DMI300B microscope (Leica Microsystems, Eindhoven, The Netherlands) over time. 

### 4.9. Statistics 

GraphPad Prism (8.2.1) was used for to perform the statistics (t-test, with significance * *p* < 0.05). 

## 5. Conclusions

CX-5461 inhibits PC cell growth in the nanomolar range and, therefore, shows promise as a PC-targeting agent. Further studies should investigate its combination with conventional DNA damaging agents, such as oxaliplatin or cisplatin. 

## Figures and Tables

**Table 1:** Sensitivity of pancreatic cancer cells to CX-5461. Each experiment was performed in triplicate and repeated at least three times. IC_50_, concentration inhibiting 50% of cell growth. SEM, standard error of the mean. Doubling time was measured in untreated cells in the exponential growth phase.

Cell Line	Average IC_50_ (nM) ± SEM	Average Doubling Time in Hours ± SEM
SUIT-2-28	77 ± 14	16.4 ± 0.20
PDAC-3	114 ± 30	18.8 ± 1.36
PANC-1	199 ± 26	28.4 ± 1.91

## Supplementary Materials

The following are available online at https://www.mdpi.com/1420-3049/24/24/4445/s1, Figure S1: Pancreatic cancer cell spheroids treated with CX-5461, Figure S2: Combination of CX-5461 and gemcitabine results in an antagonistic effect, Figure S3: CX-5461 induces mRNA expression of CDA in primary culture PDAC-3 and pancreatic cancer cells SUIT-2-28.
