# Supplementary material for: CX-5461 Inhibits Pancreatic Ductal Adenocarcinoma Cell Growth, Migration and Induces DNA Damage
Source: Molecules. 2019 Dec 4;24(24):4445. doi: 10.3390/molecules24244445 (PMC6943431; doi:10.3390/molecules24244445)
Supplement: Supplementary file 1 [file molecules-24-04445-s001.pdf]

molecules

MDPI

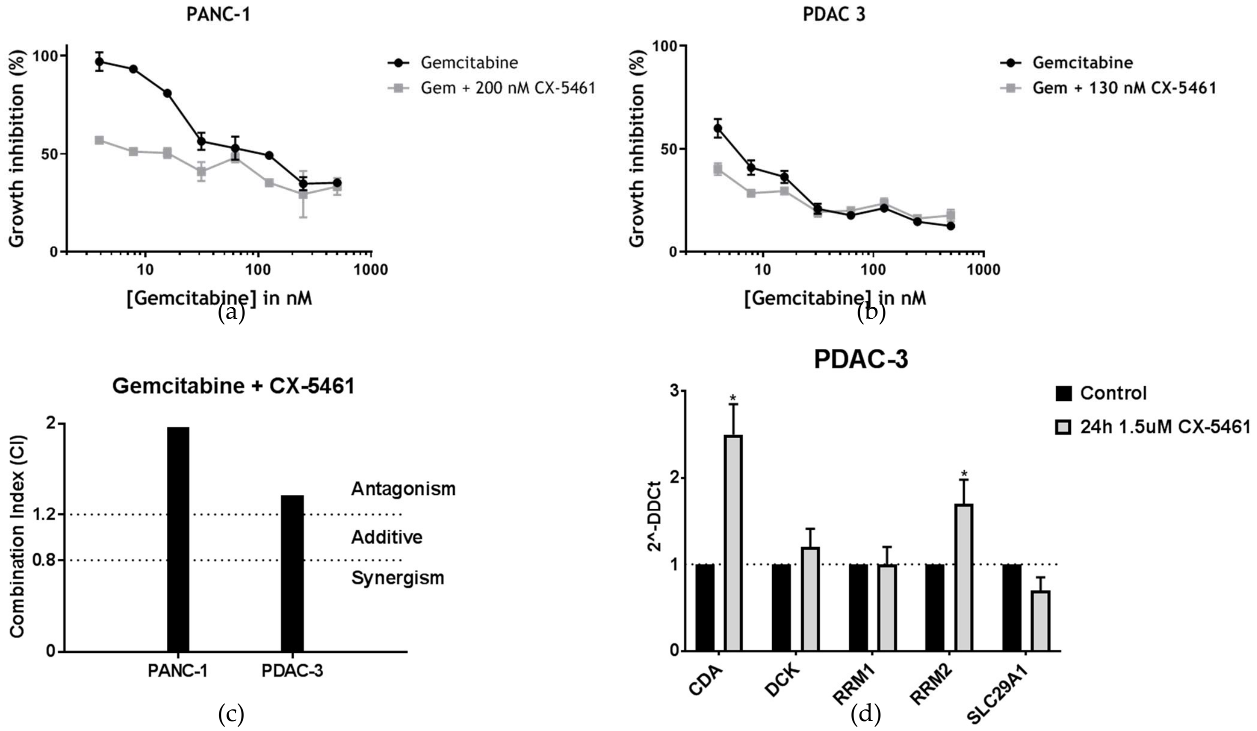

Figure S1. Combination of CX-5461 and gemcitabine results in an antagonistic effect. (a, b) Growth inhibition curves of gemcitabine and CX-5461 in PANC-1 and PDAC-3, respectively. (c) Average combination index calculated for fraction affected greater than 0.5. (d) mRNA expression of genes involved in the gemcitabine pathway.

Supplementary Materials and methods

Combination index calculation

For the drug combination studies, the SRB assay (described under materials and methods section 4.3) was used, followed by the calculation of the fraction affected (FA) and combination index (CI) as described in [23] using CalcuSyn (BioSoft, Cambridge, UK).
